# Molecular dynamics and experimental study on DAP-4/TNT melt-cast composites: interfacial interactions, structural properties, and safety performance

**DOI:** 10.3389/fchem.2026.1794947

**Published:** 2026-04-01

**Authors:** Xi Zhang, Yan Li, Binfeng Sun, Yibing Duan, Yu Qiu, Xiaoyu Shang, Fuxing Wang, Yang Liu, Lishuang Hu

**Affiliations:** 1 School of Environment and Safety Engineering, North University of China, Taiyuan, China; 2 Houma Special Machinery Factory, Houma, China; 3 Explosive Engineering and Safety Technology Research Institute of Ordnance Industry, Beijing, China

**Keywords:** interfacial interactions, melt-cast explosives, molecular dynamics, molecular perovskite energetic materials, structure–property relationship

## Abstract

To reduce the mechanical sensitivity of the high-energy molecular perovskite material DAP-4 and broaden its application scope, a DAP-4/TNT melt-cast explosive was developed using TNT as the melt-cast carrier. This study combined molecular dynamics simulation and experimental characterization to systematically investigate the interfacial interactions, multi-scale structural evolution, and overall performance of the DAP-4/TNT melt-cast explosives. Simulation results show that as the mass fraction of TNT increases, the binding energy and cohesive energy density of the system increase monotonically. At a DAP-4:TNT mass ratio of 20:80, the binding energy and cohesive energy density reach 183.55 kcal·mol^-1^ and 4.136 × 10^5^ kJ·m^-3^, respectively, with significantly improved interfacial compatibility. Experimental results indicate that TNT effectively reduces the sensitivity of DAP-4. When the TNT content increases from 60% to 80%, the impact detonation probability decreases from 50% to 28%, and the critical friction load increases from 160 N to 360 N, which is comparable to that of pure TNT. In terms of detonation velocity, the DAP-4:TNT ratio of 40:60 maintains a detonation velocity of 7201 m·s^-1^, only 8.5% lower than that of pure DAP-4, while the 20:80 ratio achieves an 18.4% increase compared to pure TNT. This work elucidates the structure-property relationship in composite energetic materials from molecular to macroscopic levels, demonstrating that rational compositional design can effectively balance energy output and safety. The findings provide a chemical engineering perspective for the development of advanced molecular perovskite energetic materials with tunable performance.

## 1 Introduction

Energetic materials are substances with specific chemical structures containing explosive functional groups in their molecules, or consist of oxidizers and combustible components in specific proportions. Under appropriate conditions, these materials can undergo self-sustaining chemical reactions and release large amounts of energy. As carriers of energy output, energetic materials play an irreplaceable role in both defense and military applications as well as civil engineering. High-energy energetic materials form the critical foundation for the development of modern weaponry, with their energy levels directly determining the destructive effectiveness and combat capabilities of munitions. Among these materials, melt-cast explosives, which are a type of mixed explosive that can be cast and assembled in a molten state, stand out due to their excellent comprehensive performance. They can perfectly adapt the loading requirements of various complex-shaped chambers, making their application value particularly significant in the military field. Melt-cast explosives are a class of mixed explosives prepared through a molten-state casting process. Their typical structure can be regarded as a special composite material system: high-energy explosives, e.g., hexogen (RDX), serve as dispersed solid particles, providing the main detonation energy for the system; while a molten carrier explosive, e.g., 2,4-dinitroanisole (DNAN), forms a continuous liquid phase that encapsulates and binds the solid particles to create a dense charge (Jing et al., 2022; Meng et al., 2020; Wang et al., 2022; Yang et al., 2019; Zhu et al., 2019). The performance of this system is influenced by multiple factors including composition ratios, interface characteristics, and process parameters. Among numerous melt-cast explosive formulations, systems using 2,4,6-trinitrotoluene (TNT) as a carrier and blending high-energy-density materials such as octogen (HMX) and hexogen (RDX) are the most widely applied. Among these, the classic Composition B explosive is prepared by high-temperature fusion of 40% TNT and 60% RDX by mass ratio (Hobbs et al., 2020; Hopler, 2011; Sarangapani et al., 2015; Taylor et al., 2006; Velicky et al., 1985), achieving a favorable balance between detonation performance and molding processability. TNT has a detonation velocity of 6970 m·s^-1^ and a detonation heat of 4148 kJ·kg^-1^. With a melting point of 80.9 °C, its relatively low melting point combined with moderate explosive properties makes it an ideal material for melt-cast explosive carriers (Wang, 2011). From the perspective of material performance, TNT has economic advantages due to its abundant raw material sources and low production costs. Its thermodynamic property, which is a melting point significantly below the thermal decomposition onset temperature (approximately 280 °C), effectively ensures the safety and feasibility of the casting process. Additionally, the chemical stability of TNT’s molecular structure renders it non-corrosive to metallic materials (Wang, 2011). It can form stable composite systems with various high-energy explosives, demonstrating excellent chemical stability and physical compatibility during long-term storage (Li et al., 2019; Ravi et al., 2011; Yuehai et al., 2017; Zhai et al., 2025).

With the continuous advancement of energetic materials research and technological innovation, various new energetic materials have emerged successively. Against this background, molecular perovskite energetic materials have gradually become a research hotspot in the field due to their unique crystal structure and excellent performance characteristics. These materials not only have favorable detonation characteristics and high thermal stability but also offer significant cost advantages. Among them, DAP-4((H_2_dabco)[NH_4_(ClO_4_)_3_]) is a representative molecular perovskite energetic material. Its excellent comprehensive properties indicate broad application prospects in the field of energetic materials (An et al., 2023; Shang et al., 2020a). Theoretical calculations indicate that DAP-4 exhibits a crystal density of 1.87 g·cm^-3^, a detonation heat of 5.87 kJ·g^-1^, a detonation pressure of 35.2 GPa, and a detonation velocity of 8.806 km·s^-1^ (Shang et al., 2020b). Current research indicates that DAP-4 exhibits exceptional thermal stability (Feng et al., 2022; Hu et al., 2021; Liang et al., 2023; Zhang et al., 2020), favorable performance characteristics, low-cost raw materials, and features a simple, easily operable preparation process. It demonstrates significant application potential in the field of energetic materials, providing an important research direction for the development and application of novel energetic materials (Chen et al., 2018; Hu et al., 2022; Yu et al., 2014). Key performance indicators of DAP-4 explosives, such as crystal density and theoretical detonation velocity, are comparable to RDX (Shang et al., 2020b), while exhibiting superior thermal stability. However, its high sensitivity and low safety threshold greatly limit its practical applications (Zhang et al., 2022).

To address the high sensitivity of DAP-4, this study adopted the formulation design approach of Composition B explosives, selecting TNT as the melt-cast carrier and compounding it with the main explosive DAP-4. By integrating molecular dynamics simulations with experimental research, we systematically investigated the interfacial interaction mechanisms, macroscopic and microscopic structural characteristics, and comprehensive performance rules of the composite system. This aims to provide theoretical foundations and technical references for the development of high-energy, low-sensitivity melt-cast explosives. This research not only expands the application potential of DAP-4 within mixed explosive systems but also provides theoretical support and experimental data for developing melt-cast explosives formulations with superior comprehensive performance.

## 2 Models and methods

### 2.1 Model construction

**FIGURE 1:**
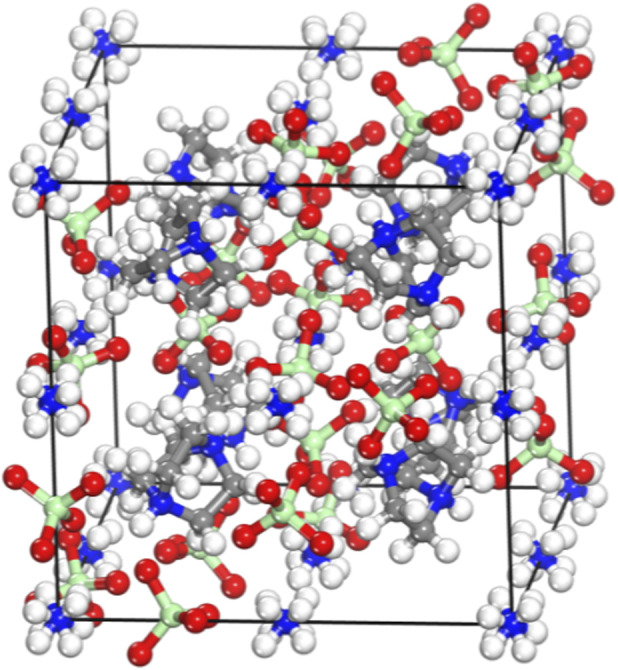
The initial unit cell structure of DAP-4.

The initial unit cell structure of DAP-4 is shown in Figure 1. This crystal belongs to the cubic crystal system with space group $Pa\bar{3}$ (No. 206), and each unit cell contains eight DAP-4 molecules. Structurally, the H_2_dabco^2+^ cation adopts a cage-like conformation, in which two nitrogen atoms are connected by three ethylene bridges, forming three six-membered rings each in a boat conformation. All nitrogen and carbon atoms in these rings exhibit sp^3^ hybridization.

**FIGURE 2:**
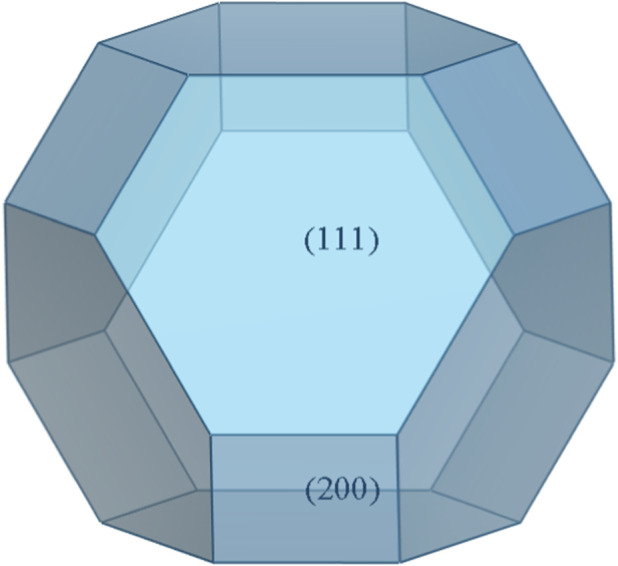
Crystal morphology of DAP-4 in vacuum.

**FIGURE 3:**
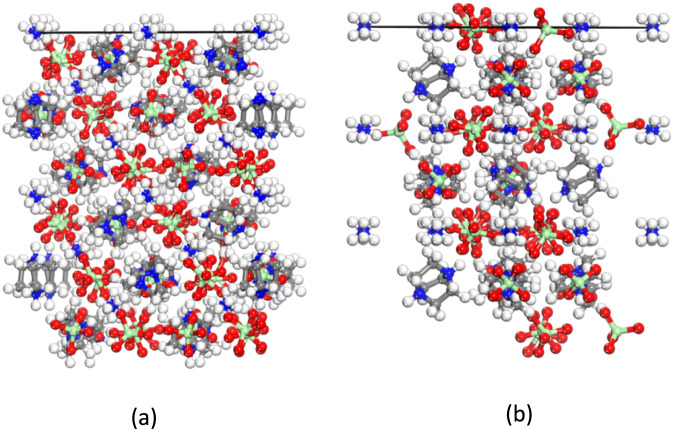
The two important crystal surfaces of DAP-4. **(a)** (1 1 1). **(b)** (2 0 0).

In the Forcite module of Materials Studio software, the geometric structure of the initial DAP-4 unit cell was optimized using the COMPASSII force field (Tao and Wang, 2017; Xiao et al., 2005; Xiao-Hong et al., 2013). Subsequently, based on the optimized structure, the Growth Morphology module simulated its crystal morphology under vacuum conditions to identify key growth planes. Relevant crystal plane parameters are listed in Table 1. Figure 2 illustrates the predicted crystal morphology of DAP-4 in vacuum. Simulation results indicate that the main exposed crystal planes of DAP-4 in vacuum are (1 1 1) and (2 0 0), and the morphology of the growth planes is shown in Figure 3. Considering that crystal planes with larger cross-sectional areas in high-energy solid-phase components are conducive to enhancing contact with the melt-cast carrier, the crystal plane with the highest proportion in vacuum is usually selected as the representative surface for subsequent analysis. Therefore, this study selected the (1 1 1) plane of DAP-4 as the main research object, which accounted for 81.39% of the crystal surface area in the vacuum environment.

**TABLE 1:** Important crystal surfaces and parameters of DAP-4.

Surface (h k L)	Multiplicity	Eatt (Total)/kcal·mol^-1^	Eatt (vdW)/kcal·mol^-1^	Total facet area/%
(1 1 1)	8	−84.71	−84.94	81.39
(2 0 0)	6	−101.60	−102.04	18.61

Using the cross-section tool in Materials Studio, the critical growth plane (1 1 1) of DAP-4 was sectioned to obtain its surface atomic structure. Subsequently, periodic cell expansion was applied to the initial cut model to construct a surface supercell of appropriate dimensions. The selection of the expansion factor required balancing two critical factors: on one hand, the surface model must contain sufficient atoms to accurately reflect the crystal-support interactions and ensure simulation reliability; on the other hand, the model size must remain within computationally manageable limits to guarantee computational efficiency. The expanded surface model obtained through the above process is ultimately defined as the DAP-4 crystal interface layer in contact with the melt-cast carrier, serving as the basis for subsequent molecular dynamics simulations.

**FIGURE 4:**
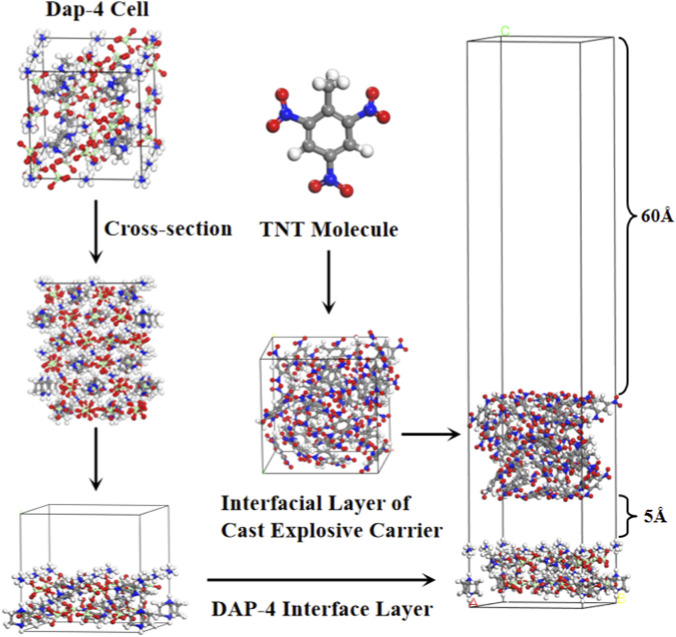
The constructure process of DAP-4/TNT interfacial model.

In the Amorphous Cell module, this study constructed an interfacial layer model for the melt-cast carrier (TNT). First, periodic boundary boxes containing the corresponding number of molecules were created based on the mass ratios and densities of each component under actual conditions, and the specific number of molecules shown in Table 2. The dimensions of the bottom surface of the box were precisely matched to those of the top surface of the DAP-4 crystal interface layer to ensure geometric consistency for subsequent interface coupling. Subsequently, geometric optimization of the constructed melt-cast carrier interface layer was performed under the COMPASSII force field until the total system energy converged to a minimum, thereby ensuring structural stability. After optimization, the melt-cast carrier interface layer was placed on top of the constructed DAP-4 crystal interface layer, with a 5 Å vacuum layer introduced between them. This distance was set based on the theoretical threshold for the effective range of van der Waals interactions, aiming to accommodate potential non-bonding interactions. To further eliminate false interactions potentially arising from periodic boundary conditions, a 60 Å vacuum layer was placed above the melt-cast carrier interface layer. Finally, all DAP-4/melt-cast carrier interface models were integrated and constructed using the Build Layers module. Figure 4 illustrates the interface model construction workflow for the DAP-4/TNT melt-cast system.

**TABLE 2:** The number of molecules in DAP-4/TNT model.

DAP-4:TNT	DAP-4 molecules	TNT molecules	Total atoms
40:60	8	23	819
30:70	8	36	1092
20:80	8	61	1617

### 2.2 Calculation methods

All molecular dynamics calculations in this chapter were performed using Materials Studio software. First, interface models of DAP-4/TNT melt-cast explosives with different mass ratios were constructed. Subsequently, structural optimization and molecular dynamics simulations were carried out sequentially in the Forcite module. During the structural optimization phase, the Geometry Optimization task was used to minimize the energy of each system, thereby obtaining stable initial configurations.

After structural optimization, the molecular dynamics simulation phase began. Simulations were conducted under the NVT ensemble, and temperature control was achieved using the Andersen thermostat method (E and Li, 2008). Non-bonding interaction parameters were configured as follows: Electrostatic interactions were calculated using the Ewald method with a precision of 0.001 kcal·mol^-1^ and a buffer width of 0.5 Å. Van der Waals interactions adopted the Atom-based method with a cutoff radius of 12.5 Å, a spline width of 1 Å, and a buffer width of 0.5 Å.

The total duration of the dynamic simulation was 500 ps, with an integration step size of 0.5 fs, corresponding to a total of 1 million steps. To ensure statistical reliability, the simulation was divided into two phases: the initial 750,000 steps (375 ps) served as the equilibration phase to allow the system to fully relax to a stable state; the subsequent 250,000 steps (125 ps) constituted the production phase for collecting trajectory data and calculating binding energy and cohesive energy density.

### 2.3 Reagents and instruments

Reagents: TNT, Hubei Dongfang Chemical Co., Ltd.; DAP-4 was synthesized in our laboratory.

Instruments: TESCAN MIRA LMS field emission scanning electron microscope, TESCAN Trading (Shanghai) Co., Ltd.; Rigaku Miniflex 600 X-ray diffractometer, Rigaku Corporation, Japan; Impact sensitivity tester, IDEA SCIENCE Technology Corporation; Friction sensitivity tester, IDEA SCIENCE Technology Corporation; Detonation velocity tester, Hunan Xiangxi QiBo Mining Instrument Factory.

### 2.4 Sample preparation

Preparation of DAP-4: First, 30 mL of deionized water was placed in a 50 mL beaker, and the beaker was secured in a heated water bath equipped with a constant-temperature magnetic stirrer. The water bath temperature was adjusted to 50 °C, and the stirring speed was set to 500 r·min^-1^ to establish a stable reaction environment. Once thermal equilibrium was reached, 2.24 g of ammonium perchlorate was added to the system and stirred continuously until completely dissolved. Subsequently, 2.35 g of triethylenediamine was introduced to the solution and stirred until fully dissolved. Using a dropping funnel, 3.26 mL of perchloric acid was added slowly at a uniform rate. After all reagents were added, the reaction mixture was maintained under constant stirring at 50 °C for 30 min to ensure complete reaction. Following the reaction, the beaker opening was sealed with sealing film, and crystallization was allowed to proceed at room temperature for 3 days. During this period, white granular crystals of DAP-4 gradually formed inside the beaker. Upon completion of crystallization, the solid product was isolated by vacuum filtration using a circulating water vacuum pump. The obtained DAP-4 granules were then dried in an oven at 50 °C for 5 h, after which they were bottled and sealed for storage.

**FIGURE 5:**
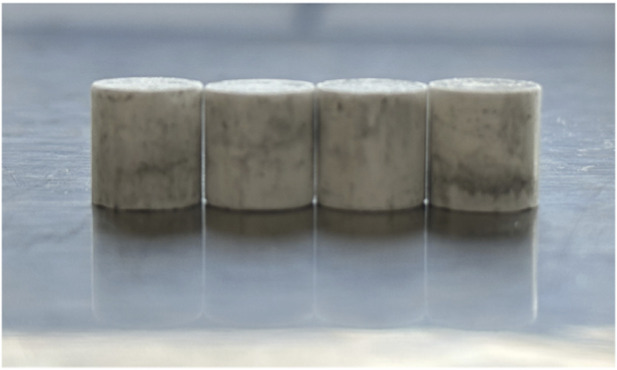
DAP-4.

To perform the blast velocity performance test, the as-prepared DAP-4 powder was compressed into cylindrical specimens with dimensions of φ10 mm × 10 mm. The macroscopic morphology of the resulting samples is presented in Figure 5.

Based on the classic formulation principles of Composition B, a DAP-4/TNT melt-blended mixture system was established with mass ratios of 40:60, 30:70, and 20:80.

**FIGURE 6:**
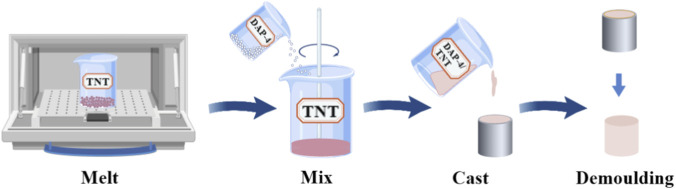
Flowchart for the preparation of DAP-4/TNT melt-cast explosive.

**FIGURE 7:**
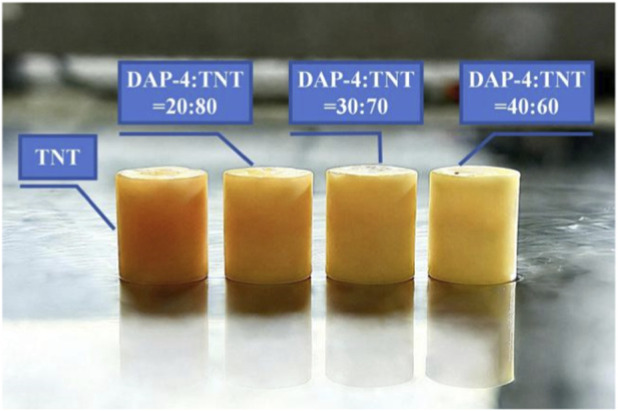
Schematic of TNT and DAP-4/TNT melt-cast explosives.

Preparation of Explosive Column Samples: Each raw material component was weighed according to the predetermined formulation ratio. Subsequently, TNT was placed in a glass beaker and heated in an oven until completely molten. After melting, DAP-4 was slowly added to the molten TNT solution while stirring continuously until thoroughly mixed. The uniformly mixed molten sample was poured into a preheated cylindrical Cr12mov mold (cavity dimensions: φ10 mm × 10 mm). After demolding, the explosive column was trimmed to the target height using a cutting tool and the bottom surface was sanded smooth with sandpaper. Finally, the dimensions of the formed explosive column were accurately measured using a vernier caliper for subsequent detonation velocity testing of this melt-cast explosive. The preparation flowchart is shown in Figure 6, and the DAP-4/TNT melt-cast explosive sample is shown in Figure 7.

Preparation of Powder Samples: The steps before casting into molds are the same as those for explosive column sample preparation. Once the mixture is thoroughly homogenized, it is placed in sample pans to cool naturally to room temperature. Subsequently, the cooled lumpy sample is ground into powder using an agate mortar for subsequent sensitivity testing and analysis of the melt-cast explosive.

The entire preparation process was conducted in a standard manner within an explosion-proof fume hood to ensure experimental safety.

### 2.5 Performance testing methods

SEM Testing: Scanning electron microscopy (SEM) was used to characterize DAP-4, TNT raw materials, and the prepared DAP-4/TNT melt-cast explosives, with an acceleration voltage of 5 KeV.

XRD Testing: An X-ray diffractometer (XRD) was used to analyze the crystal structures of DAP-4, TNT, and the prepared DAP-4/TNT melt-cast explosives. The scanning angle range was set to 10°–80°, with a copper target as the test substrate and a scanning speed of 2°·min^-1^.

Sensitivity Testing: (1) The impact sensitivity of DAP-4, TNT raw materials, and the prepared DAP-4/TNT melt-cast explosives was tested according to GJB 772A-97 Explosives Test Methods. Test conditions: 1 kg drop weight, 200 mm drop height, 20 mg charge quantity. Total test quantity: 50 charges, with 25 charges per group. (2) Friction sensitivity testing was conducted according to NATO STANAG 4487 “MIL-ST1751A: BAM Friction Test-Method 1024”. Test charge volume: (5 ± 1) mm^3^; ambient temperature: (20 ± 5) °C; relative humidity ≤80%; 6 shots per group.

Explosive Performance Testing: The detonation velocity of samples was tested in accordance with GJB 772A-97 Explosives Test Methods. The dimensions of the detonating charge column are φ10 mm × 10 mm, with a probe consisting of φ0.1 mm enameled copper wire. The test includes two sets, each involving four sample charge columns.

## 3 Results and analysis

### 3.1 Simulation results

#### 3.1.1 Bond energy analysis

The strength of interactions between components at different mixing ratios can be effectively evaluated by comparing the magnitude of binding energies. A higher binding energy indicates stronger interactions between components, resulting in a thermodynamically more stable melt-cast explosive system. Melt-cast explosives prepared at the corresponding ratio usually exhibit better overall performance. The binding energy of each component in the system can be calculated using the following formula:

$$E_{\text{bind}}=‐E\mathrm{int}=\left(E_{\text{total}}‐E_{\text{DAP}‐4}‐E_{\text{TNT}}\right)$$ (1)

In Equation 1, *E*_int_, *E*_DAP-4_, and *E*_TNT_ represent the total energy of the DAP-4/TNT melt-cast system, the energy of DAP-4, and the energy of TNT in the equilibrium system, respectively. Based on the calculation of the energy of each component at the system interface using the Forcite module, the binding energy data for DAP-4/TNT melt-cast systems with different mass ratios were obtained. The results are summarized in Table 3.

**TABLE 3:** Binding energy of DAP-4/TNT melt-cast system at different mass ratios.

DAP-4:TNT	*E*_total_/kcal·mol^-1^	*E*_DAP-4_/kcal·mol^-1^	*E*_TNT_/kcal·mol^-1^	*E*_bind_/kcal·mol^-1^
40:60	5208.75	4393.41	965.91	150.57
30:70	5530.39	4402.44	1292.90	164.94
20:80	7145.33	4412.60	2916.29	183.55

As shown in Table 3, within the DAP-4/TNT melt-cast system, the binding energy between DAP-4 and the TNT melt-cast carrier follows this order: 183.55 kcal·mol^-1^ (20:80) > 164.942 kcal·mol^-1^ (30:70) > 150.57 kcal·mol^-1^ (40:60). This indicates that as the TNT content in the system increases, the binding energy between DAP-4 and TNT gradually increases, demonstrating a continuous enhancement of the interaction between the two components. The increase in binding energy indicates more obvious intermolecular interactions between DAP-4 molecules and the TNT, leading to tighter packing of components at the microscopic scale. This arrangement facilitates reduced interfacial energy and diminished tendency toward phase separation. From a thermodynamic perspective, higher binding energy is usually associated with better component compatibility and improved system stability. This indicates that at high TNT concentrations, DAP-4 can disperse more uniformly and embed itself within the TNT melt-cast matrix, forming a composite system with a denser structure and smoother interfacial transitions. This favorable microscopic compatibility not only enhances the structural integrity of melt-cast explosives during storage and use but also provides important microscopic theoretical support for subsequent improvements of macroscopic performance.

#### 3.1.2 Cohesive energy density analysis

Cohesive energy density (CED) is commonly used to characterize the strength of intermolecular forces within condensed matter. It is defined as the energy required per unit volume for 1 mol of condensed matter to overcome intermolecular interactions during vaporization (Hang et al., 2019). Its calculation expression is given by Equation 2:

$$\text{CED}=\frac{H_{V}‐RT}{V_{m}}$$ (2)

In the equation, *H*_V_ denotes the molar heat of vaporization, *RT* represents the expansion work done during vaporization, and *V*_m_ is the molar volume. The corresponding calculation results are listed in Table 4.

**TABLE 4:** Cohesive energy density of DAP-4/TNT melt-cast system at different mass ratios.

DAP-4:TNT	van der Waals/kJ·m^-3^	Electrostatic/kJ·m^-3^	Other/kJ·m^-3^	CED/kJ·m^-3^
40:60	7.520 × 10^4^	6.052 × 10^4^	1.137 × 10^2^	1.369 × 10^5^
30:70	9.479 × 10^4^	6.711 × 10^4^	1.558 × 10^3^	1.635 × 10^5^
20:80	1.497 × 10^5^	2.602 × 10^5^	3.695 × 10^3^	4.136 × 10^5^

CED, van der Waals + Electrostatic + Other (Other represents bonding interaction energy).

Table 4 shows that in the DAP-4/TNT melt-casting system, the order of cohesive energy density between DAP-4 and the TNT melt-cast carrier is: 4.136 × 10^5^ kJ·m^-3^ (20:80) >1.635 × 10^5^ kJ·m^-3^ (30:70)>1.369 × 10^5^ kJ·m^-3^ (40:60). With the increase in TNT content, the CED of the entire system exhibits a clear upward trend. Further analysis of the components of the CED reveals that both van der Waals interactions and electrostatic interactions gradually increase with rising TNT mass fraction. This indicates an overall enhancement of intermolecular forces in the system, increasing the intermolecular energy required to overcome the transition from a condensed state to the gaseous state. In other words, the energy input needed for phase transitions or depolymerization processes increases. From the perspective of the safety and sensitivity of energetic materials, a higher CED implies tighter intermolecular bonding. Consequently, the system becomes more resistant to depolymerization or violent decomposition under external energy stimulation, indicating a gradual decrease in sensitivity. Based on the above simulation results, it can be inferred that within the composition range examined in this study, as the TNT content increases, the cohesive energy density of the DAP-4/TNT melt-cast system increases, leading to a corresponding decrease in sensitivity and an improvement in thermal safety. Therefore, under certain conditions, cohesive energy density can be used as an important theoretical indicator for qualitatively or even semi-quantitatively evaluating the sensitivity level of such melt-cast systems. It also provides valuable reference for subsequent formulation optimization and thermal stability evaluation.

### 3.2 Test results

#### 3.2.1 SEM analysis

**FIGURE 8:**
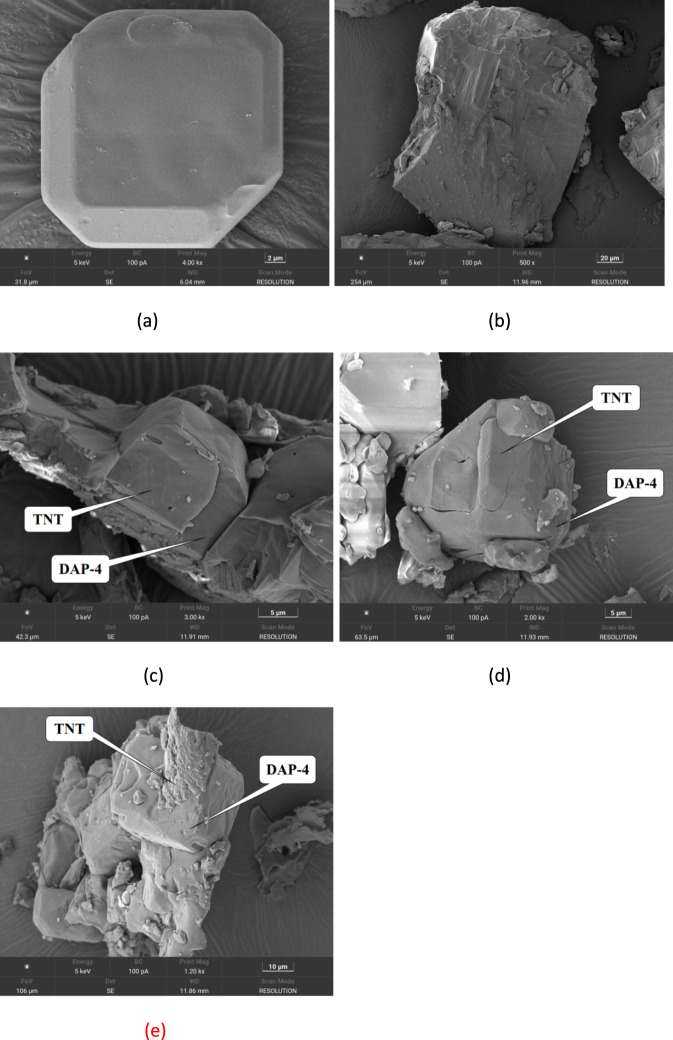
SEM images of DAP-4, TNT, and DAP-4/TNT melt-cast explosive samples with different mass ratios. **(a)** DAP-4. **(b)** Tnt. **(c)** DAP-4:TNT = 40:60. **(d)** DAP-4:TNT = 30:70. **(e)** DAP-4:TNT = 20:80.

By observing the microstructure of explosive particles, changes in the surface morphology of crystals in composite materials can be directly visualized. For this purpose, scanning electron microscopy was employed to characterize the microstructure of DAP-4, TNT, and DAP-4/TNT melt-cast explosive samples with varying mass ratios. The results are shown in Figure 8.

As shown in Figure 8a, pure DAP-4 crystals exhibit a regular block-like polyhedral structure with relatively flat and smooth surfaces, well-defined edges, and a dense crystal structure devoid of noticeable voids, indicating excellent crystallinity. In contrast, the pure TNT sample in Figure 8b has an irregular layered stacking morphology with a relatively rough surface and no regular geometric shape. Figures 8c–e show the composite microstructures obtained by melting and casting DAP-4 and TNT at different mass ratios, visually reflecting the interfacial interactions between the two materials. Figure 8c shows that when the mass ratio of DAP-4 to TNT is 40:60, TNT particles begin to adhere to the surface of DAP-4 crystals. Although DAP-4 retains a relatively intact bulk framework, initial contact between the two phases has formed at the interface. As the TNT content increases, Figure 8d shows that when the mass ratio is adjusted to 30:70, the encapsulation effect of TNT as a continuous liquid phase matrix on DAP-4 particles is significantly enhanced. The crystalline edges of DAP-4 gradually become blurred, indicating that molten TNT forms a tighter bond and fills the surface of DAP-4 during solidification. Further examination of the sample with a mass ratio of 20:80 in Figure 8e reveals that the DAP-4 crystals are now deeply embedded in the molten TNT matrix. The image demonstrates that the surface of DAP-4 is uniformly and densely encapsulated by the TNT matrix, forming a tight interfacial bond without any visible delamination or gaps. This morphological evolution is consistent with molecular dynamics simulations. The increase in binding energy confirms the enhanced intermolecular interactions between the two components from an energetic perspective, providing a microscopic theoretical basis for the effective wetting, encapsulation, and tight bonding of DAP-4 particles by the TNT melt during solidification. The densification of interfacial bonding observed by SEM directly reflects this enhanced intermolecular interaction at the macrostructural level. Together, these findings demonstrate that the system has better interfacial compatibility and structural integrity at high TNT content.

#### 3.2.2 X-ray diffraction analysis

**FIGURE 9:**
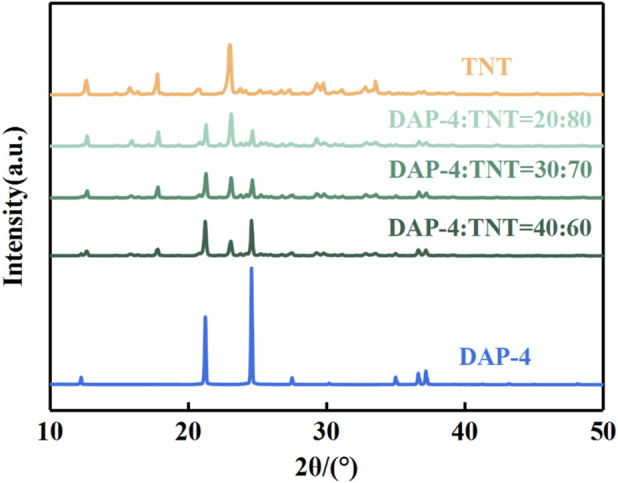
XRD images of DAP-4, TNT, and DAP-4/TNT melt-cast explosives with varying mass ratios.

To further determine the crystal structure and phase composition of the DAP-4/TNT melt-cast explosive, X-ray diffraction analysis was prepared on DAP-4, TNT, and the prepared DAP-4/TNT melt-cast explosive. The scanning angle range was set to 10°–80°, with a copper target and a scanning speed of 2°·min^-1^. The test results are shown in Figure 9.

As shown in Figure 9 based on X-ray diffraction analysis results, the experimentally synthesized DAP-4 sample exhibits characteristic diffraction peaks corresponding to the DAP-4 standard card (CCDC:152810) at 12.24°, 21.21°, 24.55°, 27.48°, 36.60°, and 37.15°, respectively. This confirms that the synthesized sample exhibits the correct crystal structure of the DAP-4 phase. Further examination of DAP-4/TNT melt-cast explosive samples at different ratios revealed XRD patterns exhibiting diffraction signals at 12.24°, 12.62°, 17.76°, 21.21°, 22.97°, 24.55°, 27.48°, 29.27°, 33.51°, 36.60°, and 37.15°. The overall diffraction pattern shows overlapping characteristic peaks of DAP-4 and TNT monomers. No diffraction peaks from unknown phases or impurities were detected, indicating high sample purity and a clear phase composition. It is worth noting that although the relative intensities of diffraction peaks differ among samples with different mass ratios, their positions at specific diffraction angles show no significant shift. This indicates that both DAP-4 and TNT retain their inherent crystal structures during the melting and casting process, with no phase transformation or significant change in lattice parameters occurring. Importantly, no diffraction peaks from new phases appear in the patterns, indicating that DAP-4 and TNT do not undergo significant chemical reactions under the process conditions. This demonstrates their excellent physicochemical compatibility, effectively preserving the intrinsic properties of DAP-4. Additionally, the reduced intensity of certain DAP-4 diffraction peaks in the melt-cast explosive may be attributed to the adhesion of TNT crystal particles onto the surface of DAP-4 crystals. This encapsulation structure creates a shielding effect on X-ray diffraction signals from specific crystal planes, consequently diminishing the intensity of corresponding diffraction peaks. These XRD test results provide strong crystallographic support for the SEM observations, offering crucial phase evidence for understanding the structural characteristics of the coexisting matrix-crystal phases in DAP-4/TNT melt-cast explosives and clarifying the microscopic mechanisms underlying their macroscopic properties.

#### 3.2.3 Mechanical sensitivity analysis

Impact sensitivity testing shall consist of 25 repetitions per group, with two parallel test groups conducted for each explosive formulation. The arithmetic mean of the explosion probabilities obtained from both groups is taken as the characteristic value of impact sensitivity for the explosive. The explosion probability is calculated using Equation 3 as follows:

$$P=\frac{X}{25}$$ (3)

The impact sensitivity and friction sensitivity test results for DAP-4, TNT, and DAP-4/TNT melt-cast explosives with different mass ratios are shown in Table 5.

**TABLE 5:** Mechanical sensitivity test results.

Sample	DAP-4	TNT	DAP-4:TNT = 40:60	DAP-4:TNT = 30:70	DAP-4:TNT = 20:80
Impact sensitivity/%	100	0	50	40	28
Friction sensitivity/N	40	360	160	360	360

According to the mechanical sensitivity test results in Table 5, both the impact sensitivity and friction sensitivity of DAP-4/TNT melt-cast explosives decrease significantly with increasing TNT content, indicating that TNT has a significant desensitizing effect on the system. Regarding impact sensitivity, pure DAP-4 has a 100% detonation probability, showing high sensitivity, while pure TNT has a 0% detonation probability, indicating excellent impact safety. When the two are mixed, the impact sensitivity of the system is between that of each component and gradually decreases as the mass fraction of TNT increases. At DAP-4:TNT ratios of 40:60, 30:70, and 20:80, the detonation probability decreases to 50%, 40%, and 28%, respectively, but remains consistently higher than that of pure TNT. This indicates that DAP-4 crystals remain a key factor influencing the impact response even at low concentrations. In terms of friction sensitivity, the critical friction load for pure DAP-4 is only 40 N, while that for pure TNT reaches as high as 360 N. As the TNT content in the melt-cast system increases from 60% to 80%, the critical friction load increases from 160 N to 360 N, significantly improving friction safety. When the TNT content reaches 70% or higher, the friction sensitivity of the system is equivalent to that of pure TNT (360 N). This macro-scale trend of blunting can be further explained by examining the binding energy and cohesive energy density derived from simulation calculations. First, the increase in binding energy indicates that DAP-4 is more firmly bound to the TNT-DAP-4 two-phase interface, enhancing the thermodynamic stability of the system. This makes it more difficult for external mechanical stimuli to initiate reactions at the interface. Second, the significant increase in cohesive energy density indicates enhanced intermolecular interactions throughout the system, hindering molecular motion. Dissociating molecules from their condensed state requires higher energy input. Therefore, the simulations reveal that the strengthened interfacial bonding and overall cohesive forces together constitute the microscopic mechanism for the reduced mechanical sensitivity of the system.

#### 3.2.4 Explosive performance analysis

**FIGURE 10:**
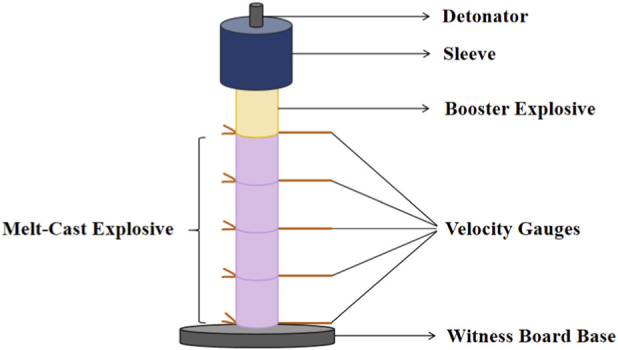
Schematic of the Detonation Velocity Test.

**FIGURE 11:**
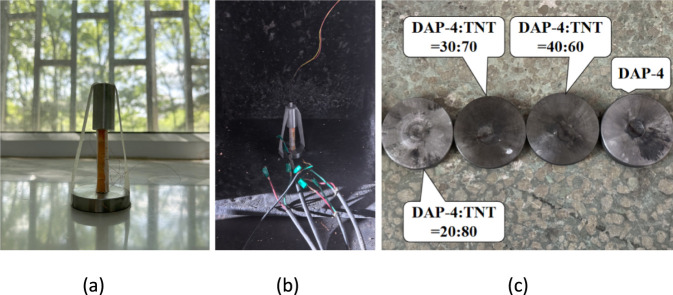
Explosive velocity test process of DAP-4 and DAP-4/TNT Melt-Cast explosive charge. **(a)** Before the test. **(b)** Test preparation. **(c)** After the test.

The schematic diagram of the detonation velocity testing apparatus used in this study is shown in Figure 10. The detonation velocity testing process for DAP-4 and DAP-4/TNT melt-cast explosives is illustrated in Figure 11.

**FIGURE 12:**
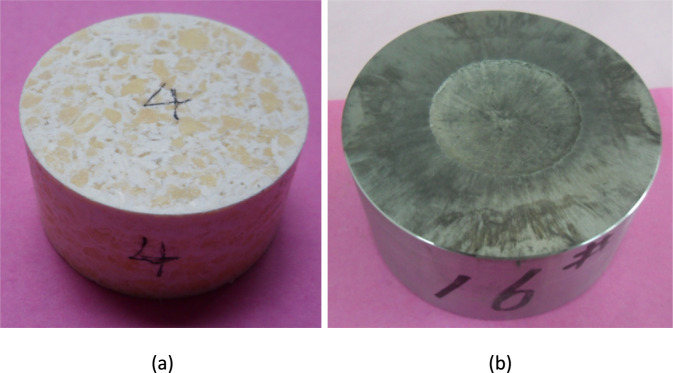
TNT charge and detonation velocity test device base. **(a)** φ40 mm TNT charge. **(b)** Base after TNT testing.

In the theory of explosive detonation, the critical detonation diameter is the minimum charge size required to maintain stable detonation. For TNT at normal density under constrained conditions, the critical detonation diameter is approximately 10 mm. When the charge diameter approaches this critical value, the propagation of the detonation wave is susceptible to boundary effects. This may lead to difficulties in initiating detonation, unstable wavefronts, or even interruption of propagation. As a result, the detonation velocity measurement data shows significant fluctuations and reduced reproducibility. The conventional test charge diameter used in this study was 10 mm, which is equivalent to the critical detonation diameter of TNT, posing a risk of unreliable results due to size criticality effects. To eliminate this influence and enhance the validity and reproducibility of the comparative experiments, additional TNT samples with a diameter of 40 mm were prepared for detonation velocity testing. This size is significantly larger than the critical diameter, ensuring a stable detonation process and obtaining reliable and reproducible reference detonation velocity values. The corresponding TNT charge column and the device base after detonation velocity testing are shown in Figure 12. The detonation velocity test results for DAP-4, TNT, and DAP-4/TNT cast explosives are presented in Table 6.

**TABLE 6:** Detonation velocity test results.

Sample	DAP-4	TNT	DAP-4:TNT = 40:60	DAP-4:TNT = 30:70	DAP-4:TNT = 20:80
Detonation velocity/m·s^-1^	7867	5104	7201	6913	6042

According to the test results in Table 6, the measured detonation velocities of pure DAP-4 and pure TNT were 7867 m·s^-1^ and 5104 m·s^-1^ respectively. The detonation velocity of DAP-4 is approximately 54.1% higher than that of TNT, indicating its superior detonation propagation capability and higher detonation performance. In the DAP-4/TNT melt-cast system, the detonation velocity shows a monotonically decreasing trend with increasing TNT mass fraction. When the mass ratio of DAP-4 to TNT is 40:60, the detonation velocity of the system reaches 7201 m·s^-1^, which is only an approximately 8.5% reduction compared to pure DAP-4. This indicates that at this mixing ratio, the detonation characteristics of the system are still mainly dominated by DAP-4. When the proportion of TNT increases to 70% and 80%, the detonation velocity decreased to 6913 m·s^-1^ and 6042 m·s^-1^ respectively, representing reductions of approximately 4% and 16.1% compared to the 40:60 sample. The detonation velocity shows a significantly greater decrease in the high TNT content range. This reflects that as the proportion of high-energy components decreases, the detonation driving capability and reaction rate of the system weaken, leading to a corresponding reduction in the stable propagation velocity of the detonation wave. Notably, even a small amount of DAP-4 exhibits a significant reinforcing effect on TNT-based systems. For example, the sample with a DAP-4:TNT ratio of 20:80 achieves a detonation velocity of 6042 m·s^-1^, which is an approximately 18.4% increase compared to pure TNT. This indicates that in TNT-dominant formulations, the incorporation of high-energy components can enhance the energy release intensity in the reaction zone and improve the detonation wave coupling conditions. This promotes the detonation waves to approach a more stable propagation state, thereby maintaining detonation velocities above those achievable with pure TNT.

As observed in Figures 11c, 12b, obvious erosion pits are formed at the center of the component bases after detonation velocity testing, and the severity of the pits increases with higher DAP-4 content in the formulation. Specifically, the central pit in the base of the DAP-4:TNT = 20:80 sample is shallow with relatively diffuse boundaries. As the DAP-4 content increases to 30% and 40%, the central pit gradually deepens and becomes more concentrated, with clearer radial erosion traces around it. The pure DAP-4 sample shows the most pronounced erosion on the base, indicating its stronger impact and penetration effects on the component base. This phenomenon indicates that as the proportion of DAP-4 increases, the detonation energy release intensity and shock load level of the system increase, intensifying the transient erosion and plastic deformation effects of detonation products on the component base. Therefore, the depth of the central pit on the component base tends to increase with the increase in DAP-4 content. This morphological result is consistent with the trend shown in Table 6, where the detonation velocity increases with DAP-4 content, providing intuitive verification for the detonation velocity test conclusions.

Figure 12 shows the morphology of the φ40 mm TNT sample and its accessory base after detonation velocity testing. Considering the critical diameter effect of TNT, detonation is more likely to propagate at the boundary, leading to fluctuations or even lower detonation velocities. Using larger-diameter samples for comparative testing helps reduce the influence of geometric scale on detonation stability, thereby enhancing the reproducibility and comparative validity of TNT reference data. Consequently, the TNT detonation velocity values in Table 6 can be regarded as comparative results obtained under more stable detonation conditions, making them more suitable for performance comparisons with the DAP-4 and DAP-4/TNT systems.

## 4 Conclusion

1. Molecular dynamics simulation results indicate that both the binding energy and cohesive energy density of the DAP-4/TNT system exhibit a monotonically increasing trend with increasing TNT mass fraction. At a 20:80 ratio, the binding energy increases to 183.55 kcal·mol^-1^, and the cohesive energy density reaches 4.136 × 10^5^ kJ·m^-3^, which are 1.22 times and 3.02 times those of the 40:60 ratio system, respectively. The significant increase in binding energy reflects enhanced molecular interactions between DAP-4 and TNT, contributing to improved interfacial compatibility and suppressed phase separation tendency. The increase in cohesive energy density provides a microscopic mechanism for reduced mechanical sensitivity at the energy level. These simulation results are highly consistent with the densely packed coating structure of TNT on DAP-4 particles and the stable coexistence of both phases observed in SEM and XRD experiments, collectively confirming the critical role of TNT in enhancing the interfacial stability of the composite system.
2. Mechanical sensitivity testing confirms that TNT significantly reduces the sensitivity of DAP-4. As the TNT mass fraction increases from 60% to 80%, the impact detonation probability decreases from 50% to 28%, and the critical friction load increases from 160 N to 360 N, reaching a level comparable to that of pure TNT. This trend of improved macroscopic safety is consistent with the increased cohesive energy density revealed by molecular dynamics simulations. Higher TNT content strengthens the intermolecular forces throughout the system, thereby suppressing the formation of “hot spots” under mechanical stress and effectively improving the safety of DAP-4/TNT melt-cast explosives.
3. Detonation velocity test results indicate that the detonation performance of the DAP-4/TNT system exhibits systematic variation with component ratio. When the mass ratio of DAP-4 to TNT is 40:60, the detonation velocity of the system reaches 7201 m·s^-1^, which is only an 8.5% decrease compared to pure DAP-4, demonstrating excellent high-energy characteristics. Even at 80% TNT content, the detonation velocity of the system reaches 6042 m·s^-1^, which is an 18.4% increase compared to pure TNT. This confirms the significant detonation-enhancing effect of DAP-4 on the TNT matrix. These results demonstrate that synergistic optimization of detonation performance and safety properties can be achieved through rational control of the composition ratio. Among them, the sample with a 30:70 ratio achieves a good balance between detonation velocity and mechanical sensitivity, with a detonation velocity of 6913 m·s^-1^ and an impact detonation probability of 40%, along with a friction load of 360 N, showing potential application prospects.

## Data Availability

The original contributions presented in the study are included in the article/supplementary material, further inquiries can be directed to the corresponding authors.
